# Sequential organ failure assessment score predicts mortality after coronary artery bypass grafting

**DOI:** 10.1186/s12893-017-0219-9

**Published:** 2017-03-06

**Authors:** Chih-Hsiang Chang, Shao-Wei Chen, Pei-Chun Fan, Cheng-Chia Lee, Huang-Yu Yang, Su-Wei Chang, Heng-Chih Pan, Feng-Chun Tsai, Chih-Wei Yang, Yung-Chang Chen

**Affiliations:** 1 Kidney Research Center, Department of Nephrology, Chang Gung Memorial Hospital, Linkou Medical Center, Taoyuan, Taiwan; 2 Department of Cardiothoracic and Vascular Surgery, Chang Gung Memorial Hospital, Linkou Medical Center, Taoyuan, Taiwan; 3 Department of Nephrology, Chang Gung Memorial Hospital, Keelung branch, New Taipei City, Taiwan; 4 Clinical Informatics and Medical Statistics Research Center, College of Medicine, Chang Gung University, Taoyuan, Taiwan; 5 School of medicine, College of Medicine, Chang Gung University, 199 Tung Hwa North Road, Taoyuan, Taipei 105 Taiwan

**Keywords:** Coronary artery by pass grafting, Sequential organ failure assessment, Society of thoracic surgeons mortality risk, Mortality, Cardiorenal syndrome

## Abstract

**Background:**

Mortality after coronary artery bypass grafting (CABG) is generally associated with underlying disease and surgical factors overlooked in preoperative prognostic models. Sequential Organ Failure Assessment (SOFA) and Acute Physiology and Chronic Health Evaluation (APACHE II) scores are widely used in intensive care units for outcome prediction. This study investigated the accuracy of these models in predicting mortality.

**Methods:**

Between January 2010 and April 2013, 483 patients who underwent isolated CABG were enrolled. The clinical characteristics, outcomes, and prognostic model scores of the patients were collected. Discrimination was assessed using the area under the curve approach.

**Results:**

Both SOFA and APACHE II scores were effective for predicting in-hospital mortality. Among the organ systems examined in the SOFA, the cardiac and renal systems were the strongest predictors of CABG mortality. Multivariate analysis identified only the SOFA score as being an independent risk factor for mortality.

**Conclusion:**

In summary, the SOFA score can be used to accurately identify mortality after isolated CABG.

## Background

Studies have reported that the mortality of cardiac surgery ranges from 2.94 to 32.5 according to different surgeries and population [1–3]. Prognostic models developed by the Society of Thoracic Surgeons European System for Cardiac Operative Risk Evaluation for mortality and morbidity are widely used before operations. However, unexpected intraoperative factors might influence the outcomes of these patients. General severity scoring systems, such as the Acute Physiology and Chronic Health Evaluation (APACHE II) [4] and Sequential Organ Failure Assessment (SOFA) [5], are generally used in intensive care units (ICUs) to predict mortality, but their validity in isolated coronary artery bypass grafting (CABG) patients is not well established [6], Therefore, we undertook a post hoc analysis of a prospectively accumulated database to evaluate the accuracy of the SOFA and APACHE II for predicting postoperative mortality on the date of surgical ICU admission.

## Methods

### Study participants and data collection

This clinical study was conducted in full compliance with the ethical principles of the Declaration of Helsinki and was consistent with Good Clinical Practice guidelines and the applicable local regulatory requirements. The local institutional review board of Chang Gung Memorial Hospital approved our study protocol. The requirement for obtaining patient consent was waived because the study utilized prospectively collected data for post hoc analysis. This database was constructed for medical quality assurance. Patients of ages older than 20 years were enrolled in this study. Between January 2010 and April 2013, 483 consecutive patients who received isolated CABG in a single tertiary referral hospital were investigated. Pertinent data on patients’ medical history including clinical characteristics, demographic data, and a scoring system were extracted from this database. Congestive heart failure (CHF, based on Framingham criteria and defined using New York Heart Association classifications), shock (defined as hypotension with systolic arterial blood pressure of 90 mmHg despite adequate fluid resuscitation), and myocardial infarction (MI; defined according to the 2007 Expert Consensus Document of Circulation from the *European Heart Journal*) were evaluated. Illness severity was assessed using APACHE II and SOFA [4, 5] scores determined on the day of ICU admission. The primary outcome was in-hospital mortality. Patients who underwent CABG received standard medical care according to the 2011 American College of Cardiology Foundation/American Heart Association guidelines for CABG [7].

### Statistical analysis

The Kolmogorov–Smirnov test was used to determine the normal distribution for each variable. Student’s *t* test was used to compare the means of continuous variables and normally distributed data; otherwise, the Mann–Whitney *U* test was used. Categorical data were tested using the chi-square test or Fisher exact test. Furthermore, the Hosmer–Lemeshow goodness-of-fit test was used for calibration when evaluating the number of observed and predicted deaths for the entire range of death probabilities. Discrimination was assessed using the area under the curve (AUC), which was compared using a nonparametric approach [8]. Cumulative survival curves as a function of time were generated using the Kaplan–Meier approach and compared using a log-rank test. All statistical tests were 2-tailed; a value of *P* < .05 was considered statistically significant.

## Results

### Study population characteristics

The 483 patients were all adults and had a mean age of 62.9 years; of them, 17.6% (*n*  =  85) were female. Overall in-hospital mortality was 9.9%. All patient characteristics are listed in Table 1. Compared with the survival group, the mortality group was older, was more likely to have end-stage renal disease (ESRD), and was more likely to be on ventilator support before surgery. Those who died also had higher creatinine (Cr), lower albumin (ALB), and lower hemoglobin (Hb) levels and poorer preoperative heart conditions such as greater more preoperative intra-aortic balloon pumping (IABP) usage, extracorporeal membrane oxygenation (ECMO) usage, more shock, more recent MI, and a lower ejection fraction. Table 2 lists the surgical details and postoperative data of the patients. The nonsurvivors required the surgery more urgently and had more intraoperative IABP and ECMO usage compared with the survivors. They also had a greater proportion of shock as well as higher Cr, and lower Hb levels after the operation. ICU stay was markedly prolonged in the mortality group.

**Table 1:** Preoperative demographic data and clinical characteristics of survival and mortality groups (Expressed as Mean ± Standard Error)

	All Patients (*n* = 483)	Survival (*n* = 435)	Mortality (*n* = 48)	*p*-value
*Preoperative demographic data*				
Age (years)	62.9 ± 0.5	62.3 ± 0.5	68.8 ± 1.5	<0.001
Sex, female (%)	85 (17.6)	75 (17.2)	10 (20.8)	0.535
Diabetes mellitus (%)	252 (52.2)	223 (51.3)	29 (60.4)	0.228
Hypertension (%)	376 (77.8)	340 (78.2)	36 (75.0)	0.617
ESRD (%)	57 (11.8)	43 (9.9)	14 (29.2)	<0.001
Shock (%)	66 (13.7)	42 (9.7)	24 (50.0)	<0.001
Mechanical ventilation, *n* (%)	45 (9.3)	33 (7.6)	12 (25.0)	<0.001
Serum Creatinine (mg/dL)	2.0 ± 0.12	1.79 ± 0.11	3.85 ± 0.51	<0.001
Albumin (g/L)	3.85 ± 0.03	3.89 ± 0.30	3.25 ± 0.93	<0.001
Hemoglobin (g/dL)	12.5 ± 0.1	12.7 ± 0.1	10.8 ± 0.3	<0.001
hs-CRP (mg/L)	26.4 ± 2.7	23.1 ± 2.7	58.6 ± 16.3	<0.001
*Preoperative heart condition*				
CHF Fc III/IV (%)	77 (15.9)	63 (14.5)	14 (29.2)	0.008
IABP, (%)	60 (12.4)	40 (9.2)	20 (41.7)	<0.001
ECMO, (%)	11 (2.3)	7 (1.6)	4 (8.3)	0.017
Ejection fraction (%)	53 ± 1	54 ± 1	40 ± 2	<0.001
Recent MI, (%)	225 (46.6)	191 (43.9)	34 (70.8)	0.001
CAD vessels	2.80 ± 0.02	2.8 ± 0.02	2.8 ± 0.07	0.437
*Preoperative scores*				
STS-risk of mortality	7.7 ± 0.6	5.4 ± 0.5	27.9 ± 3.1	<0.001
EuroSCORE II	6.8 ± 0.4	5.4 ± 0.4	18.9 ± 2.1	<0.001

*Abbreviations*: *CAD* coronary artery disease, *CHF Fc* congestive heart failure functional class, *ECMO* Extracorporeal Membrane Oxygenation, *ESRS* End stage renal disease, *EuroSCORE* European System for Cardiac Operative Risk Evaluation, *hs*-*CRP* high-sensitivity C-reactive protein, *IABP* intra-aortic balloon pump, *ICU* intensive care unit, *MI* myocardial infarction, *NS* not significant, *STS* Society of Thoracic Surgeons

**Table 2:** Postoperative outcomes and clinical characteristics of survival and mortality patients (Expressed as Mean ± Standard Error)

	All Patients (*n* = 483)	Survival (*n* = 435)	Mortality (*n* = 48)	*p*-value
*Surgical detail*				
Urgent operation (%)	124 (25.7)	91 (20.9)	33 (68.8)	<0.001
On pump (%)	332 (68.7)	293 (67.4)	39 (81.3)	0.049
On pump-clamp time (minutes)	-	89.7 ± 3.4	77.8 ± 11.4	-
On pump-bypass time (minutes)	-	115.8 ± 2.7	135.5 ± 11.1	-
Bypass graft number	2.98 ± 0.04	2.91 ± 0.04	2.69 ± 0.09	0.076
*Postoperative data*				
IABP, (%)	111 (23.0)	95 (21.8)	16 (33.3)	0.072
ECMO, (%)	26 (5.4)	11 (2.5)	15 (31.3)	<0.001
Shock	143 (29.7)	105 (24.2)	38 (79.2)	<0.001
Bilirubin Total (units/L)	1.18 ± 0.04	1.16 ± 0.04	1.29 ± 0.14	0.195
Serum Creatinine (mg/dL)	1.82 ± 0.1	1.68 ± 0.10	3.32 ± 0.41	<0.001
Hemoglobin (g/dL)	10.6 ± 0.1	10.7 ± 0.1	9.5 ± 0.3	<0.001
*Postoperative scores*				
SOFA	6.6 ± 0.1	6.2 ± 0.1	10.8 ± 0.4	<0.001
APACHE II	12.9 ± 0.3	12.0 ± 0.2	21.1 ± 0.9	<0.001
*Patient outcome*				
ICU stay (day)	4.9 ± 0.4	4.4 ± 0.4	10.3 ± 1.6	<0.001

*Abbreviations*: *ECMO* Extracorporeal Membrane Oxygenation, *IABP* intra-aortic balloon pump, *SOFA* Sequential Organ Failure Assessment

### Scoring systems and in-hospital mortality

**Fig. 1:**
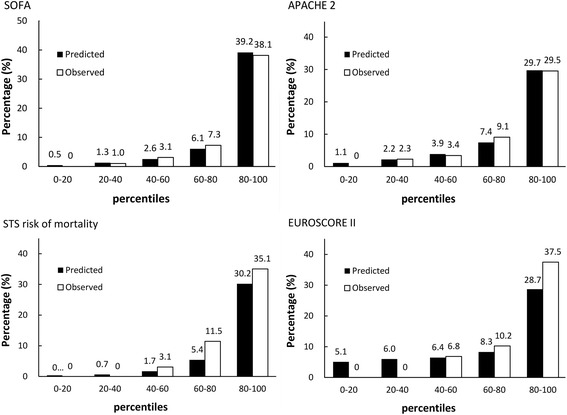
Observed and expected (mean) mortality rates divided by quintiles of expected mortality calculated using the STS risk model, EuroSCORE II, SOFA, and APACHE II

The mean Society of Thoracic Surgeons (STS) mortality risk, European System for Cardiac Operative Risk Evaluation (EuroSCORE) II, SOFA, and APACHE II scores were 7.7, 6.8, 6.6, and 12.9, respectively. All scores differed significantly between the survivors and nonsurvivors. The AUC results listed in Table 3 indicate that the SOFA, STS mortality risk, APACHE II, and EuroSCORE II all had satisfactory discriminatory ability (0.912 ± 0.019, *P* < .001, 0.898 ± 0.017, *P* < .001, 0.866 ± 0.027, *P* < .001, and 0.863 ± 0.022, *P* < .001). Both the SOFA and STS mortality risk were significantly superior to EuroSCORE II (*P* = .016 and *P* < .001, respectively). Low *P* values for the Hosmer–Lemeshow test indicated that there were no statistical hints against an assumed fit of the scoring system. Both the postoperative SOFA and APACHE II scores exhibited close agreement between the observed and expected mortality. Figure 1 presents the observed and predicted mortality results for the scoring systems stratified into quintiles.

**Table 3:** Calibration and discrimination results for the scoring methods in predicting mortality

	Calibration			Discrimination		
	goodness-of-fit (*χ* ^2^)	df	*p*	AUC ± SE	95% CI	*p*
Postoperative SOFA	3.361	6	0.762	0.912 ± 0.019	0.875 – 0.949	<0.001
Postoperative APACHE II	6.335	8	0.610	0.866 ± 0.027	0.813 – 0.919	<0.001
STS-risk of mortality	29.654	8	<0.001	0.898 ± 0.017	0.864 – 0.932	<0.001
EuroSCORE II	40.472	8	<0.001	0.863 ± 0.022	0.821 – 0.906	<0.001

*Abbreviations*: *APACHE* Acute Physiology and Chronic Health Evaluation, *AUC* area under the curve, *CI* confidence interval, *df* degree of freedom, *EuroSCORE* European System for Cardiac Operative Risk Evaluation, *SE* standard error, *SOFA* Sequential Organ Failure Assessment, *STS* Society of Thoracic Surgeons

To investigate the individual organ systems that affect post-CABG mortality, we dissected the SOFA score components to calculate the discriminatory power of each (Table 4). The cardiovascular system followed by the renal system had the higher discriminatory power, after which were coagulation and neurology, both with similar discriminatory power. Liver and respiration had the lowest discriminatory power among the individual factors. Combining the cardiovascular and renal system components resulted in increased discriminatory power, but this power was still less than that of the entire SOFA score (*P* < .001).

**Table 4:** Calibration and discrimination results for each sofa parameter in predicting mortality

	Calibration			Discrimination		
	goodness-of-fit (*χ* ^2^)	df	*p*	AUC ± SE	95% CI	*p*
Respiration	6.006	1	0.014	0.529 ± 0.046	0.439 – 0.619	0.524
Renal	16.566	2	<0.001	0.803 ± 0.033	0.738 – 0.867	<0.001
Liver	0.421	1	0.516	0.578 ± 0.048	0.484 – 0.673	0.087
Cardiovascular	2.947	3	0.400	0.805 ± 0.034	0.737 – 0.872	<0.001
Coagulation	2.929	1	0.087	0.677 ± 0.047	0.584 – 0.770	<0.001
Neurological	5.550	1	0.018	0.703 ± 0.048	0.608 – 0.797	<0.001
Cardiovascular + Renal	2.303	4	0.680	0.871 ± 0.023	0.825 – 0.917	<0.001

*Abbreviations*: *AUC* area under the curve, *CI* confidence interval, *df* degree of freedom, *SE* standard error

**Fig. 2:**
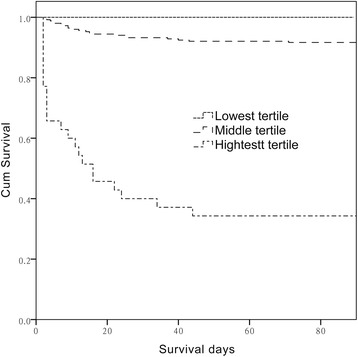
Kaplan–Meier survival curves of mortality with log-rank test results according to SOFA score tertiles

Figure 2 illustrates the cumulative survival rate, stratified according to SOFA score tertiles. Patients in the high and middle tertiles had significantly lower survival rates than did those in the low tertile (log-rank *P* < .001)

### Logistic regression analysis for mortality according to postoperative variables

According to univariable and multivariable logistic regression models based on patient characteristics and postoperative factors, only the age, postoperative ECMO and SOFA score were independently associated with mortality (Table 5).

**Table 5:** Logistic regression analysis of postoperative factors for mortality

Parameter	Beta Coefficient	Standard error	Odds ratio (95% CI)	*p*
*Univariate logistic regression*				
Age	0.052	0.016	1.053 (1.022–1.086)	0.001
Diabetes mellitus	0.300	0.312	1.350 (0.732–2.489)	0.336
Hypertension	0.294	0.357	0.342 (0.667–2.699)	0.409
ESRD	1.207	0.356	3.342 (1.662–6.719)	0.001
Serum Creatinine	0.211	0.054	1.235 (1.111–1.372)	<0.001
Hemoglobin	−0.412	0.100	0.662 (0.545–0.805)	<0.001
IABP	0.576	0.330	1.779 (0.923–3.394)	0.081
ECMO	2.962	0.459	13.343 (7.876–47.560)	<0.001
SOFA	0.737	0.151	2.090 (1.556–2.807)	<0.001
*Multivariate logistic regression*				
Age	0.062	0.021	1.064 (1.020–1.110)	0.004
ECMO	2.298	0.678	9.959 (2.636–37.620)	0.001
SOFA	0.711	0.097	2.036 (1.685–2.460)	<0.001
constant	−12.623	1.851	-	-

*Abbreviations*: *ECMO* Extracorporeal Membrane Oxygenation, *IABP* intra-aortic balloon pump, *SOFA* Sequential Organ Failure Assessment

## Discussion

This present investigation examined 483 isolated CABG patients admitted to an ICU after operation. The overall in-hospital mortality rate was 9.9%, which was higher than that reported in previous studies. Tamayo et al. studied post-cardiac surgery patients and found a 90-day in-hospital mortality rate of 8.8% [9]. Curiel-Balsera et al. performed a study using the ARIAM Andalus adult cardiac surgery registry, which included data from 11 hospitals in the autonomous region of Andalusia between 2008 and 2011, and reported intra-ICU mortality of 7.7% and 30-day mortality of 9.3% [10]. Rodríguez-Rieiro et al. reported an observed mortality rate of 7.69% [11]. Our preoperative STS and EuroSCORE II scores were much higher than those reported in the literature [12–14]. Potential explanations for this are as follows. First, the current data were collected in the largest tertiary referral hospital in Northern Taiwan, in which a large proportion of patients have advanced comorbidities such as ESRD (11.8%), which is well known to be a high-risk population for surgical mortality [15]. Second, in traditional Chinese culture, families typically seek treatment for patients even when doing so involves high surgical risk and a high likelihood of complications [16]. Third, of the patients who underwent emergent and salvage CABG, some had severe preoperative cardiogenic shock (12.4%) and received mechanical circulatory support (MCS), namely patients with IABP (13.2%) and ECMO (2.5%), both of which are positively associated with CABG mortality. Early mortality in patients undergoing emergent and salvage CABG is substantial, with the in-hospital mortality rates of 13 and 41%, respectively [17]. Patients requiring preoperative MCS have operative mortality of 37.2%, and those undergoing CABG as a salvage procedure have an operative mortality of 53.3% [18]. Finally, the low total hospital costs (approximately 10% of the costs in the United States) and large reimbursement amounts under the health insurance system of Taiwan may have affected the quality of patient care and negatively affected the CABG outcomes [19].

Numerous prognostic risk models for cardiac surgery have been introduced into current practice. Among them, the EuroSCORE, published in 1999 [20]; revised EuroSCORE II, published in 2012 [21]; and STS score, published in 2008 [22] are widely used. These assessments, which are based on collected data, can be used to predict the risk of cardiac surgery mortality according to patient demographics and clinical variables, and can be determined using an easy-to-use online calculator. No previous study has compared preoperative and postoperative risk modes for predicting the occurrence of mortality in isolated CABG. Our data showed that preoperative scores underestimated mortality in high-risk patients (Fig. 1). Thus, a postoperative score is necessary for ICU doctors to reevaluate condition severity. In this study, we demonstrated that SOFA and APACHE II scores at ICU day 1 have similar predictive effectiveness for mortality. SOFA score based upon respiratory, renal, hepatic, cardiovascular, neurological and coagulation systems, were used as an assessment tool to evaluate outcome. The Cardiac Surgery Score (CASUS) was designed for predicting cardiac surgery mortality by using 10 variables measured upon ICU admission [23]. Several studies have concluded that the scale can effectively predict cardiac surgery mortality, and have recommended its use [24]. The CASUS also evaluates several organ functions and has similar parameters to those of SOFA. However, lactic acid was not routinely recorded postoperatively in our ICU; thus, we could not validate CASUS results in this study. The Post Cardiac Surgery (POCAS) prognostic score was developed on the basis of 4 parameters (mean arterial pressure [MAP], lactate, bicarbonate, and international normalized ratio [INR]), and has favorable discrimination in predicting postcardiac mortality [9]. Although we did not compare POCAS and SOFA scores, MAP and lactate represent the cardiac system, bicarbonate represents the renal system, and the INR represents coagulation and the liver system, which might explain why the SOFA and POCAS results were similar.

Patients receiving CABG mainly exhibit 2 complex cardiorenal syndromes. First, patients with coronary arterial disease are usually older and have DM, HTN, and CHF, which cause chronic kidney disease (CKD) through microvascular and macrovascular pathophysiology. Moreover, sympathetic tone, renin–angiotensin–aldosterone system activation, and endothelin release cause excess vasoconstriction and impair glomerular filtration. CKD also induces erythropoietin deficiency, decreased vitamin D receptor activation, and fluid acumination, which might affect cardiac function through cardiomyopathy. Second, during CABG, patients experience low cardiac output, nonpulsatile blood flow, hemolysis, and free iron release, which induce oxidative stress and reactive oxygen species and impair kidney function. Otherwise, excess diuretic and analgesic use might result in acute kidney injury (AKI). In fact, AKI affects 12–30% of patients undergoing CABG, contributes to increased in-hospital mortality and reduced long-term survival, and results in high medical expenditure, CKD, and dialysis dependence [25–28]. Postoperative AKI not only contributes to increased in-hospital mortality and reduced long-term survival but also results in high medical expenditure, CKD, and dialysis dependence. In the present study, the mortality group patients exhibited anemia, hypoalbuminemia, and poor renal function, which reflect the severity of heart failure, water retention, inflammation, and malnutrition and which are known risk factors for mortality after cardiac surgery [14, 29, 30]. The occurrence of cardiac and renal dysfunction varies among patients admitted to the ICU with CABG, with different degrees of association existing between individual organ system failure and ICU mortality [31]. This explains why combining the cardiac and renal factors of the SOFA score is an accurate technique for assessing mortality in patients who receive CABG.

### Study limitations

Despite the favorable results obtained in this study, some crucial limitations must be noted. First, our study was limited by its post hoc analysis nature and relatively small number of cases from a single referral center in Asia; care should be used when generalizing the results to different populations. Second, scoring was performed only on the first day of ICU admission. The sequential application of these scoring systems (for example, daily or weekly) may reflect dynamic aspects of clinical diseases and thus provide more complete data on mortality risk. Finally, the etiology of mortality is often multifactorial, and postoperative care factors that were not included in the scoring systems may have affected the prediction results.

## Conclusions

The analysis results highlight the strong discriminative power of the SOFA and APACHE II scores in predicting mortality in patients after CABG. Among the different components of the SOFA, renal dysfunction is the only one that is also incriminated in cardiac failure, indicating that mortality is often part of a cardiorenal syndrome. The multivariate analysis identified the SOFA score alone as an independent risk factor for mortality. Higher SOFA scores are correlated with higher mortality. In conclusion, SOFA might be the most effective tool for guiding the use of preventive and early therapeutic strategies to prevent mortality and improve clinical outcomes for patients who undergo CABG. Future studies could conduct multicenter and repeated measurements to enhance accuracy for future clinical management.
